# Fluoxetine attenuates hepatic ischemia–reperfusion injury through antioxidant, anti-inflammatory and anti-apoptotic mechanisms

**DOI:** 10.1007/s00210-026-05563-8

**Published:** 2026-06-19

**Authors:** Musab Işık, Gülderen Şahin, Gülnaz Kervancıoğlu, Muhittin Onur Yaman, Nermin Yelmen, İbrahim Güner

**Affiliations:** 1 Department of Physiology, Faculty of Medicine, Istanbul Aydın University, Istanbul, Turkey; 2 Department of Histology and Embryology, Faculty of Medicine, Istanbul Health and Technology University, Istanbul, Turkey; 3 Department of Medical Services and Techniques, Istanbul University–Cerrahpaşa Vocational School of Health Services, Istanbul, Turkey; 4 Department of Physiology, Faculty of Medicine, Tekirdağ Namık Kemal University, Tekirdağ, Turkey

**Keywords:** Apoptosis, Fluoxetine, Hepatic ischemia–reperfusion injury, Inflammation, Oxidative stress

## Abstract

Hepatic ischemia–reperfusion injury is a major clinical problem associated with liver surgery, trauma, and transplantation, characterized by oxidative stress, inflammation, microcirculatory dysfunction, and apoptosis. Fluoxetine, a selective serotonin reuptake inhibitor, has been reported to exert antioxidant, anti-inflammatory, and antiapoptotic effects. The present study aimed to investigate the potential protective effects of fluoxetine against experimental hepatic ischemia–reperfusion injury in rats. Male Wistar rats were randomly assigned to three groups: control, ischemia–reperfusion and fluoxetine-treated ischemia–reperfusion (*n* = 7 each). Hepatic ischemia–reperfusion injury was induced by infrarenal abdominal aortic cross-clamping for 60 min followed by 120 min of reperfusion. Fluoxetine (20 mg/kg/day, intraperitoneal) was administered for three consecutive days prior to ischemia. Oxidative stress markers, antioxidant parameters, inflammatory and anti-inflammatory cytokines, apoptotic markers, and specific tissue injury biomarkers were measured in liver homogenates using ELISA. Histopathological alterations were evaluated by light microscopy. Ischemia–reperfusion significantly increased oxidant markers, inflammatory cytokines, NF-κB activation, apoptotic indices, liver enzyme levels, and histological damage, while reducing antioxidant capacity. Fluoxetine treatment markedly restored antioxidant defenses, suppressed oxidative stress, inflammation, apoptosis, and microcirculatory injury, and significantly improved histopathological findings compared with the untreated ischemia–reperfusion group. Fluoxetine exerts a protective effect against hepatic ischemia–reperfusion injury through antioxidant, anti-inflammatory and antiapoptotic mechanisms, suggesting its potential as a therapeutic agent in conditions associated with hepatic ischemia–reperfusion.

## Introduction

The liver is the largest parenchymal organ and gland in the body, representing nearly 2.5% of adult body weight with an average mass of approximately 1500 g. It performs numerous vital functions, primarily including nutrient metabolism and the elimination of waste products, and serves as the first central organ through which venous blood from the intestines passes before entering the systemic circulation. Complete loss of hepatic function can result in fatal outcomes within minutes. Owing to its unique regenerative capacity, the liver is able to restore its functional mass following tissue loss; while hepatocytes in the resting state exhibit limited proliferative activity under physiological conditions, their proliferative capacity markedly increases in response to reductions in organ volume or functional impairment, thereby ensuring the structural and functional continuity of the liver. The primary role of the liver is to regulate the passage of substances absorbed from the digestive tract before they enter the systemic circulation and to ensure their biological safety. In this context, the liver carries out a wide range of critical functions, including the synthesis and secretion of bile, bilirubin metabolism, regulation of carbohydrate, lipid, and protein metabolism, and the metabolism, conjugation, and excretion of endogenous and exogenous substances. Additionally, the liver plays a central role in the storage of various minerals—particularly iron and copper—as well as vitamins A, D, E, K, and B12, and glycogen. Its endocrine functions include the activation of vitamin D, conversion of thyroxine (T4) to triiodothyronine (T3), synthesis of angiotensinogen, and metabolism of various hormones. Moreover, the liver is a potent immunological organ. Through the reticuloendothelial system, it filters bacteria and particulate matter from portal blood, performs phagocytosis via Kupffer cells, participates in antigen presentation, and contributes to the clearance of hemolysis products. Collectively, these multifaceted functions underscore the liver’s indispensable role in maintaining metabolic, hematological, immunological, and endocrine homeostasis, rendering it essential for survival (Ozougwu et al. 2017; Konishi et al. 2017; Hoekstra et al. 2013).

Hepatic ischemia–reperfusion (IR) injury is a critical pathophysiological process that occurs during the restoration of blood flow following partial or complete interruption of hepatic perfusion and directly affects organ viability. During surgical procedures such as major tumor resections, liver trauma, vascular reconstructions, and liver transplantation, the duration of ischemia may be prolonged, leading to metabolic disturbances caused by insufficient oxygen and nutrient supply to hepatocytes. In many hepatic surgeries, particularly in severe trauma and the resection of large intrahepatic lesions, the Pringle maneuver is frequently employed to control bleeding by temporarily interrupting hepatic blood inflow, which inevitably results in hepatic ischemia. During the subsequent reperfusion phase, the re-establishment of blood flow and oxygen delivery paradoxically exacerbates ischemia-initiated cellular injury and markedly increases tissue damage. Hepatic IR injury is classified into warm and cold ischemia; warm ischemia occurs during trauma, shock, and elective liver surgeries, whereas cold ischemia develops during organ preservation prior to transplantation. Hepatic ischemia–reperfusion injury remains a major clinical challenge and is recognized as one of the leading causes of liver dysfunction and graft failure following transplantation, major hepatectomy, and hemorrhagic shock. The pathophysiology of hepatic IR injury is highly complex and involves mitochondrial dysfunction, oxidative stress, dysregulated cell death, excessive activation of the immune system, inflammatory disturbances, and impairment of the hepatic microcirculation. These pathological processes contribute to hepatocellular injury, multiple organ dysfunction, poor clinical prognosis, and increased mortality. Hepatic IR injury is also closely associated with graft dysfunction, impaired liver function, and early transplant failure, accounting for a substantial proportion of unsuccessful transplant outcomes. In addition, biological factors such as age and sex influence susceptibility to hepatic IR injury, with more severe damage observed in males. Given the increasing clinical burden of ischemic and inflammatory liver disorders, there is a growing need to better understand the underlying mechanisms of hepatic IR injury and to develop effective therapeutic strategies (Cursio et al. 2015; Montalvo-Jave et al. 2008; Serracino-Inglott et al. 2001; Klune et al. 2010; Zhao et al. 2025; Abu‐Amara et al. 2010; Huang et al. 2025; Abdelnaser et al. 2026).

Fluoxetine is a selective serotonin reuptake inhibitor (SSRI) that regulates serotonergic neurotransmission in the central nervous system and is widely used in clinical practice. It is commonly prescribed for the treatment of depression, obsessive–compulsive disorder, bulimia nervosa, panic disorder, and anxiety disorders (Erman et al. 2015; Altan et al. 2023; Mohamed Kamel et al. 2021; Caiaffo et al. 2016). Fluoxetine undergoes extensive hepatic metabolism, primarily yielding its active metabolite norfluoxetine, along with several other metabolites, which are subsequently eliminated via the urinary route (Ganguly et al. 2022; Djordjevic et al. 2011). Fluoxetine demonstrates pronounced pleiotropic biological activities beyond its serotonergic antidepressant properties, including the regulation of inflammatory, oxidative, and apoptotic pathways. The current evidence suggests that fluoxetine enhances antioxidant defense systems and anti-inflammatory cytokine profiles while concurrently inhibiting pro-inflammatory mediators, pro-oxidant enzyme expression, and free radical generation. These coordinated effects indicate that its therapeutic benefits are largely mediated through multifaceted cellular modulatory mechanisms within both central and peripheral tissues. Experimental studies have demonstrated that fluoxetine administration suppresses edema in a dose-dependent manner, suggesting that it may serve as a potential therapeutic agent in the prevention and treatment of inflammatory processes. In this context, fluoxetine has been reported to inhibit the release of pro-inflammatory cytokines, reduce the expression of pro-oxidant enzymes and free radicals (including hydroxyl and superoxide anion radicals), and enhance in vivo antioxidant defense mechanisms by reversing oxidative damage. Moreover, fluoxetine has been shown to attenuate oxidative cellular injury, suppress apoptosis, and increase the levels of antioxidant enzymes such as superoxide dismutase, catalase, and glutathione peroxidase. Consistent with these properties, fluoxetine exerts antioxidant, anti-inflammatory, and antiapoptotic effects by downregulating pro-inflammatory markers while upregulating anti-inflammatory markers (Caiaffo et al. 2016; Erman et al. 2015; Altan et al. 2023; Irfan et al. 2024; Kubera et al. 2010; Ghosh et al. 2020).

The primary rationale for investigating the effects of fluoxetine in an experimental model of hepatic IR injury in this study was its potential to modulate the inflammatory response, oxidative stress, and microcirculatory function. Fluoxetine may attenuate inflammatory injury by suppressing NF-κB activation, thereby reducing the production of pro-inflammatory cytokines such as IL-6, TNF-α, and IL-1β, as well as limiting neutrophil infiltration. In addition, through its antioxidant properties, fluoxetine may increase GSH and SOD levels while decreasing lipid peroxidation, thus mitigating oxidative stress. Furthermore, fluoxetine may enhance microcirculatory perfusion during reperfusion by improving blood flow via serotonin- and endothelial nitric oxide synthase (eNOS)–mediated mechanisms. Within this context, the present study aimed to investigate the potential protective effects of fluoxetine against hepatic IR injury. To the best of our knowledge, this is the first experimental study demonstrating the protective effects of fluoxetine in hepatic ischemia–reperfusion injury.

## Materials and methods

### Chemicals

All chemicals of the highest analytical grade were obtained from Sigma Chemical Co. (St. Louis, MO, USA). Deionized water was used for all analyses. All reagents were stored at + 4 °C in accordance with the manufacturers’ instructions and allowed to equilibrate to room temperature prior to use.

### Animals and ethical approval

**Fig. 1:**
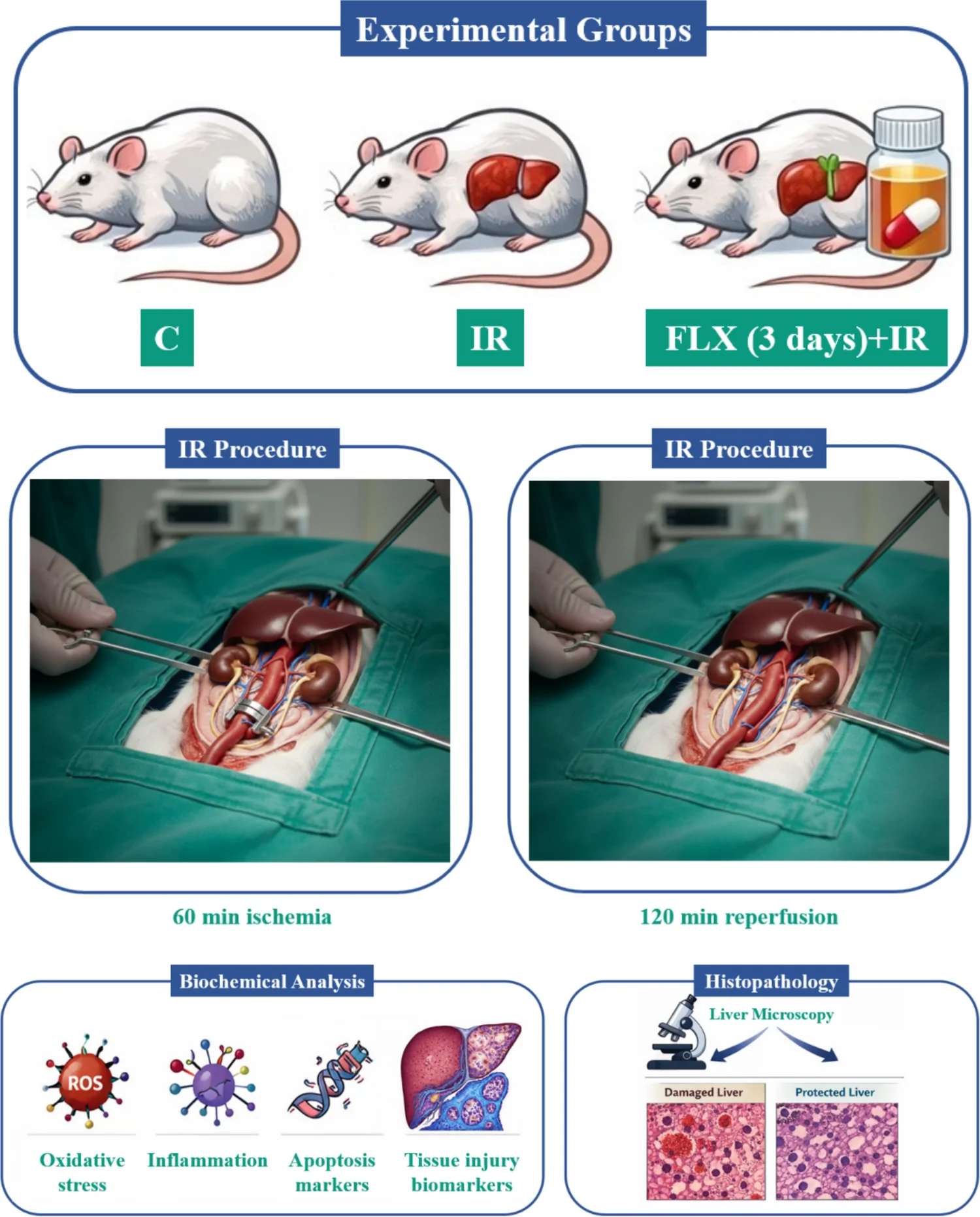
Graphical overview of the experimental design (This figure was generated using an artificial intelligence–based image generation tool and is intended for illustrative purposes only)

In this study, male Wistar rats weighing 350–400 g were used. All experimental animals were housed in transparent polycarbonate cages under standard laboratory conditions with a 12-h light/12-h dark cycle, a controlled temperature of 22 ± 2 °C, and relative humidity of 45–50%. The rats were provided with standard pellet chow and had free access to tap water. All experimental procedures were conducted in accordance with the National Institutes of Health (NIH) Guide for the Care and Use of Laboratory Animals and were approved by the Istanbul University Experimental Animals Ethics Committee (approval no. 2012/48). The rats were randomly allocated into three groups: a saline-treated control group (C), a saline-treated ischemia–reperfusion group (IR), and a fluoxetine-treated ischemia–reperfusion group (FLX + IR), with seven animals in each group (*n* = 7). Rats in the FLX + IR group received fluoxetine at a dose of 20 mg/kg/day via intraperitoneal injection once daily for three consecutive days prior to ischemia surgery (Fig. 1). In a previous ischemia study, fluoxetine administered at a dose of 20 mg/kg was shown to exert significant protective effects against cellular damage and to enhance antioxidant activity. Therefore, this dose was selected in the present study to ensure comparability with the existing experimental literature that has reported similar protective effects using comparable dosing regimens. A 3-day pre-treatment protocol with fluoxetine (20 mg/kg, i.p.) has been widely used in experimental ischemia–reperfusion models (Kim et al. 2007).

### Experimental design and surgical procedures

Anesthesia was induced by intraperitoneal administration of pentobarbital sodium (60 mg/kg) in the IR and FLX + IR groups. Spontaneous respiration was maintained via a polyvinyl chloride tube inserted through a tracheostomy. Throughout the surgical procedures, the rats’ body temperature was kept constant at 37 ± 0.5 °C using a heating pad. For hemodynamic monitoring, blood pressure was continuously measured by catheterization of the carotid artery with a 22-gauge catheter. Following aseptic preparation of the skin, a midline incision was made to perform laparotomy. To prevent fluid loss during the experiment, prewarmed physiological saline was administered into the peritoneal cavity. For exposure of the infrarenal abdominal aorta, the intestinal loops were gently displaced to the left using moistened gauze pads. After careful dissection and isolation of the vessel, ischemia was induced by applying an aortic cross-clamp to the infrarenal abdominal aorta for 60 min. The infrarenal abdominal aorta served exclusively as the site of ischemia induction, whereas all biochemical and histopathological analyses were performed on liver tissue samples (Vascu-Statts II, midi flat 1001–532; Scanlan Int., St Paul, MN, USA). To minimize heat loss and tissue desiccation, the abdominal cavity was covered with humidified gauze. Following completion of the ischemic period, the clamp was removed and reperfusion was initiated for 120 min. The adequacy of both occlusion and reperfusion was assessed by observing pulsations distal to the clamped aortic segment and was further confirmed by continuous blood pressure monitoring. After completion of the ischemia–reperfusion protocol, euthanasia was performed under deep pentobarbital sodium anesthesia (150 mg/kg, IP). For histopathological evaluation, a portion of the samples was fixed in formalin, while the remaining specimens were stored at − 80 °C for biochemical analyses (Altan et al. 2023; Sínay et al. 2008; Kim et al. 2007) (Fig. 1). For biochemical analyses, approximately 190–200 mg of liver tissue was weighed and homogenized in ice-cold 20 mM Tris–HCl buffer (pH 7.4) at a ratio of 20% (w/v) using a Bosch Scintilla SA homogenizer (Switzerland). The homogenates were centrifuged at 5000 × g for 10 min, and biochemical parameters were determined in the supernatant fractions. All chemicals of the highest analytical grade were obtained from Sigma Chemical Co. (St. Louis, MO, USA). Deionized water was used for all analyses. All reagents were stored at + 4 °C in accordance with the manufacturers’ instructions and allowed to equilibrate to room temperature prior to use.

### Biochemical analyses by ELISA

All samples were analyzed in duplicate using rat-specific commercially available ELISA kits validated for use in tissue homogenates. Prior to analysis, kits and reagents were equilibrated to room temperature in accordance with the manufacturer’s instructions. Standard curves were generated for each assay to ensure linearity and analytical reliability. Intra- and inter-assay coefficients of variation were within the acceptable ranges specified by the manufacturer. Measured values were normalized to total protein content in each sample.

### Measurement of oxidant and antioxidant parameters

Oxidative and antioxidant markers, including superoxide dismutase (SOD), glutathione (GSH), lipid hydroperoxides (LOOH), malondialdehyde (MDA), total oxidant status (TOS), total antioxidant status (TAS), and reactive oxygen species (ROS) were measured by ELISA employing commercially available assay kits from eBioscience (San Diego, CA, USA), as per the manufacturer’s recommended procedures.

### Measurement of pro-inflammatory and anti-inflammatory cytokines

In liver tissue homogenates, the levels of pro-inflammatory cytokines, including tumor necrosis factor-alpha (TNF-α), interleukin-1 beta (IL-1β), and interleukin-6 (IL-6), as well as the anti-inflammatory cytokine interleukin-10 (IL-10), were measured using commercial ELISA kits from eBioscience (San Diego, CA, USA), following the manufacturers’ instructions.

### Measurement of apoptotic and other parameters

The concentrations of nuclear factor kappa B (NF-κB), matrix metalloproteinase-9 (MMP-9), caspase-9 (Casp-9), 8-hydroxy-2′-deoxyguanosine (8-OHdG), nitric oxide (expressed as nitrite/nitrate), and hyaluronan (HA) were determined by enzyme-linked immunosorbent assay (ELISA) using commercially available kits supplied by Abcam (Cambridge, MA, USA), following the manufacturer’s instructions.

### Histopathological examination

Liver tissues obtained from rats in the C, IR, and FLX + IR groups were fixed in 10% buffered neutral formalin and embedded in paraffin following routine tissue processing. Sections of 4 μm thickness were cut from the paraffin blocks and stained with hematoxylin and eosin. The prepared sections were examined under a light microscope (Olympus CX-31, Shinjuku-ku, Tokyo, Japan), and images were captured using a digital camera (Olympus LC20, Shinjuku-ku, Tokyo, Japan); representative photomicrographs were obtained from each slide for comparative purposes. Histopathological assessment focused on the presence of hydropic swelling, granular degeneration, microvesicular vacuolization, focal necrosis, and disruption of hepatic cord architecture in the liver parenchyma; inflammation and fibrosis in the portal areas; and hyperemia within the sinusoids. Histopathological changes were evaluated semi-quantitatively using the scoring system described by Suzuki, with grades defined as 0 (no injury), 1 (minimal), 2 (mild), 3 (moderate) and 4 (severe injury) (Gedik et al. 2008; Suzuki et al. 1993). All sections were assessed independently by two blinded observers who were unaware of the experimental groups. Inter-observer consistency was taken into consideration during the evaluation process to ensure reliability of the scoring results.

### Statistical analyses

Data are presented as mean ± standard error of the mean (SEM). Statistical analyses were performed using GraphPad Prism version 5.0 (GraphPad Software, San Diego, CA, USA). Differences among groups were analyzed by one-way analysis of variance (ANOVA), and when statistical significance was detected, multiple comparisons were carried out using the Bonferroni post hoc test. A p value of < 0.05 was considered statistically significant for all analyses.

## Results

### Oxidant/anti-oxidant levels and liver function tests

**Fig. 2:**
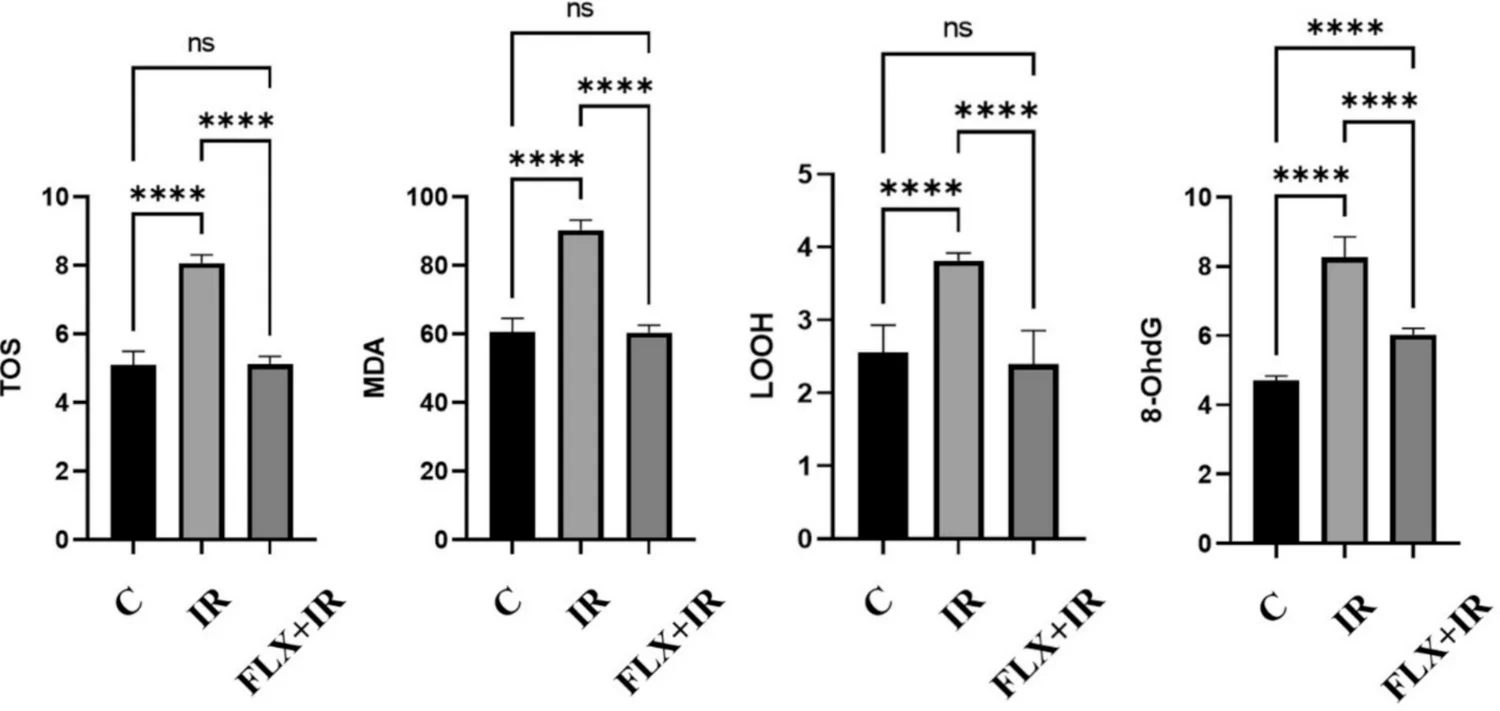
Effects of fluoxetine on oxidative stress parameters in hepatic ischemia–reperfusion injury. The IR group demonstrated significantly increased oxidative stress markers compared with the control group. Fluoxetine treatment significantly reduced TOS, MDA, LOOH, and 8-OHdG levels compared with the untreated IR group. Statistical significance: ^*^p < 0.05, ^**^p < 0.01, ^***^p < 0.001, ^****^p < 0.0001

**Fig. 3:**
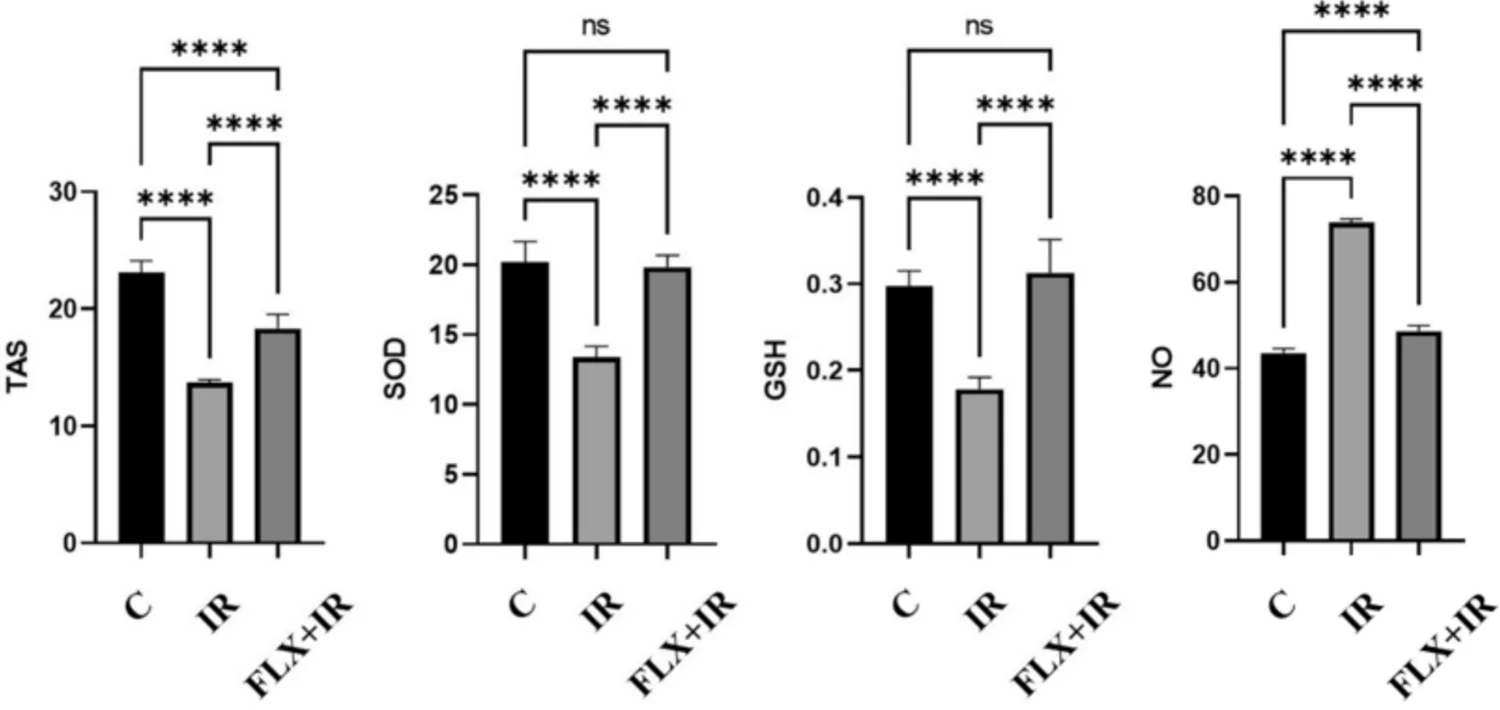
Effects of fluoxetine on antioxidant defense parameters. hepatic ischemia–reperfusion significantly decreased antioxidant markers including TAS, SOD, GSH and NO compared with the control group. Fluoxetine administration significantly restored antioxidant capacity in the FLX + IR group compared with the untreated IR group. Statistical significance: *p < 0.05, **p < 0.01, ***p < 0.001, ****p < 0.0001

**Fig. 4:**
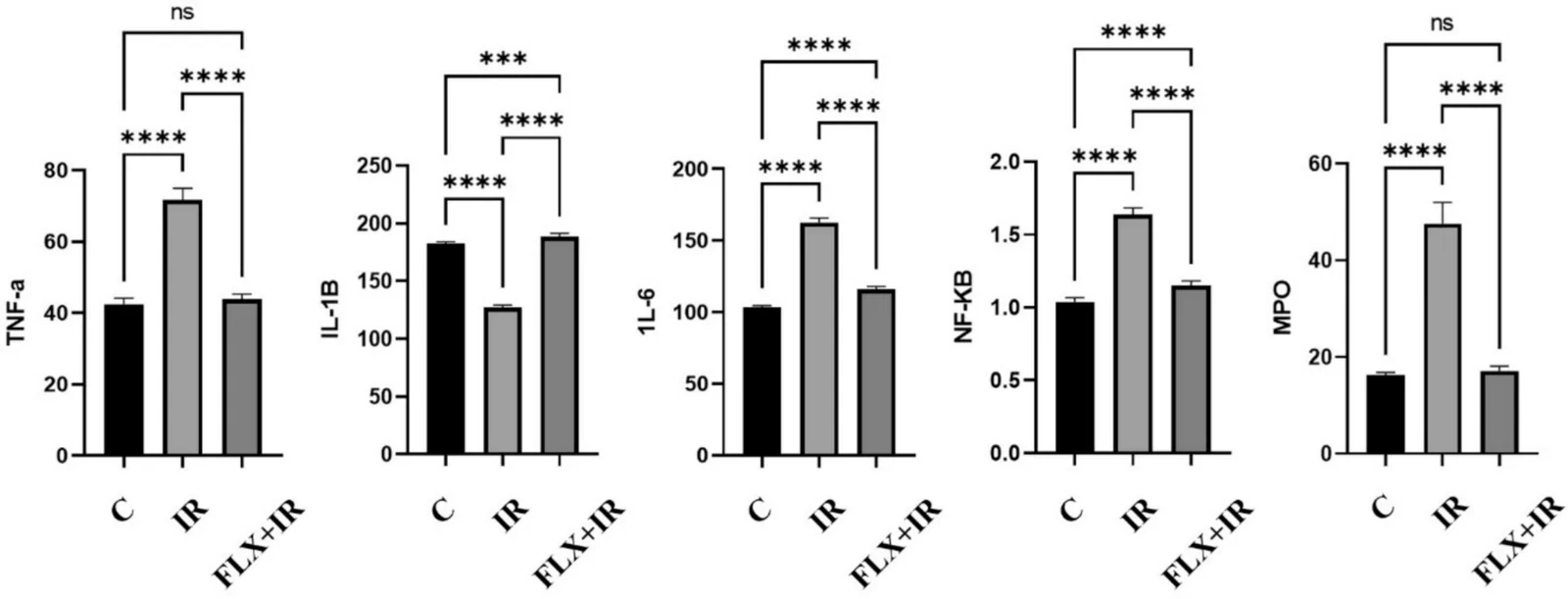
Effects of fluoxetine on inflammatory mediators in hepatic ischemia–reperfusion injury. The IR group showed significantly increased levels of pro-inflammatory cytokines (TNF-α, IL-1β, IL-6), MPO activity, and NF-κB activation compared with the control group. Fluoxetine treatment significantly suppressed these inflammatory responses compared with the untreated IR group. Statistical significance: *p < 0.05, **p < 0.01, ***p < 0.001, ****p < 0.0001

**Fig. 5:**
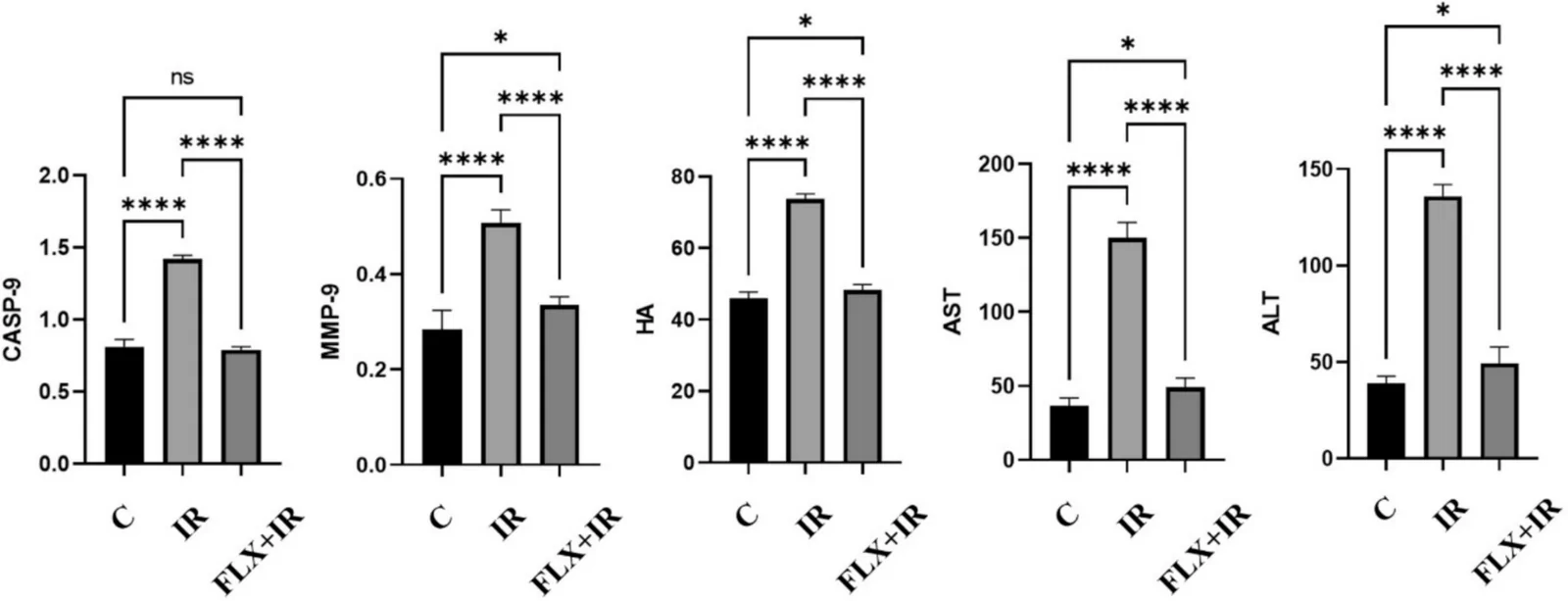
Effects of fluoxetine on apoptotic and liver injury-related biomarkers. hepatic ischemia–reperfusion significantly increased apoptotic marker caspase-9 and liver injury markers including ALT, AST, MMP-9, and hyaluronic acid (HA). Fluoxetine treatment significantly attenuated these increases, indicating reduced hepatocellular damage. Statistical significance: *p < 0.05, **p < 0.01, ***p < 0.001, ****p < 0.0001

SOD activity (U/wet tissue) was 20.18 ± 1.49 in the control group and decreased significantly to 13.39 ± 0.77 in the IR group compared with controls (*p* < 0.0001). In the FLX + IR group, SOD activity increased significantly to 19.84 ± 0.85 compared with the IR group (*p* < 0.0001). No statistically significant difference was observed between the control and FLX + IR groups (*p* > 0.05). Reduced GSH levels (µmol/wet tissue) were determined as 0.29 ± 0.017 in the control group, 0.17 ± 0.013 in the IR group, and 0.31 ± 0.038 in the FLX + IR group. GSH levels were significantly decreased in the IR group compared with the control group (*p* < 0.0001). In the FLX + IR group, GSH levels were significantly higher than those in the IR group (*p* < 0.0001). No statistically significant difference was detected between the control and FLX + IR groups (*p* > 0.05). LOOH levels (nmol/wet tissue) were 2.55 ± 0.37 in the control group, 3.81 ± 0.10 in the IR group, and 2.39 ± 0.46 in the FLX + IR group. LOOH levels were significantly increased in the IR group compared with the control group (*p* < 0.0001). In contrast, LOOH levels were significantly reduced in the FLX + IR group compared with the IR group (*p* < 0.0001). No significant difference was observed between the control and FLX + IR groups (*p* > 0.05). TAS levels (U/100 mg protein) were 23.10 ± 1.00 in the control group, 13.72 ± 0.25 in the IR group, and 18.31 ± 1.21 in the FLX + IR group. TAS levels were significantly decreased in the IR group compared with the control group (*p* < 0.0001). Although TAS levels were significantly increased in the FLX + IR group compared with the IR group (*p* < 0.0001), they remained significantly lower than those in the control group (*p* < 0.0001). TOS levels (nM/100 mg protein) were 5.09 ± 0.39 in the control group, 8.06 ± 0.23 in the IR group, and 5.12 ± 0.22 in the FLX + IR group. TOS levels were significantly elevated in the IR group compared with the control group (*p* < 0.0001). In the FLX + IR group, TOS levels were significantly reduced compared with the IR group (*p* < 0.0001), with no significant difference observed relative to the control group (*p* > 0.05). MDA levels (nM/100 mg protein) were 60.46 ± 4.11 in the control group, 90.24 ± 2.99 in the IR group, and 60.35 ± 2.17 in the FLX + IR group. MDA levels were significantly increased in the IR group compared with the control group (*p* < 0.0001). Treatment with fluoxetine significantly reduced MDA levels compared with the IR group (*p* < 0.0001), with no statistically significant difference observed between the control and FLX + IR groups (*p* > 0.05). MPO activity (μU/g tissue) was 16.29 ± 0.51 in the control group, 47.62 ± 4.42 in the IR group, and 17.14 ± 0.97 in the FLX + IR group. MPO activity was significantly increased in the IR group compared with the control group (*p* < 0.0001). In the FLX + IR group, MPO activity was significantly decreased compared with the IR group (*p* < 0.0001), with no significant difference relative to the control group (*p* > 0.05). ROS levels (U/100 mg protein) were 3943.87 ± 297.39 in the control group, 5669.48 ± 168.88 in the IR group, and 4351.48 ± 73.74 in the FLX + IR group. ROS levels were significantly elevated in the IR group compared with the control group (*p* < 0.0001). Although ROS levels were significantly reduced in the FLX + IR group compared with the IR group (*p* < 0.0001), they remained significantly higher than those in the control group (*p* < 0.01). ALT levels (U/L) were 38.96 ± 3.66 in the control group, 135.97 ± 5.94 in the IR group, and 49.31 ± 8.49 in the FLX + IR group. ALT levels were significantly increased in the IR group compared with the control group (*p* < 0.0001). In the FLX + IR group, ALT levels were significantly reduced compared with the IR group (*p* < 0.0001), although they remained significantly higher than those in the control group (*p* < 0.05). AST levels (U/L) were 36.39 ± 5.52 in the control group, 150.28 ± 10.24 in the IR group, and 49.13 ± 6.28 in the FLX + IR group. AST levels were significantly elevated in the IR group compared with the control group (*p* < 0.0001). Fluoxetine treatment significantly decreased AST levels compared with the IR group (*p* < 0.0001); however, AST levels remained significantly higher than those in the control group (*p* < 0.05); (Table 1), (Figs. 2, 3, 4, 5).

**Table 1:** Comparative results of oxidant/antioxidant levels and liver function tests among the experimental groups

Groups	Control group	IR group	FLX + IR group
	Oxidant/anti-oxidant levels and liver function tests		
SOD (U/wet tissue)	20.18 ± 1.49	13.39 ± 0.77^aaaa^	19.84 ± 0.85^bbbb^
GSH (µmol/wet tissue)	0.29 ± 0.017	0.17 ± 0.013^aaaa^	0.31 ± 0.038^bbbb^
LOOH (nmol/wet tissue)	2.55 ± 0.37	3.81 ± 0.10^aaaa^	2.39 ± 0.46^bbbb^
TAS (U/100 mg protein)	23.10 ± 1.00	13.72 ± 0.25^aaaa^	18.31 ± 1.21^aaaa, bbbb^
TOS (nM/100 mg protein)	5.09 ± 0.39	8.06 ± 0.23^aaaa^	5.12 ± 0.22^bbbb^
MDA (nM/100 mg protein)	60.46 ± 4.11	90.24 ± 2.99^aaaa^	60.35 ± 2.17^bbbb^
MPO (µU/g tissue)	16.29 ± 0.51	47.62 ± 4.42^aaaa^	17.14 ± 0.97^bbbb^
ROS (U/100 mg protein)	3943.87 ± 297.39	5669.48 ± 168.88^aaaa^	4351.48 ± 73.74^aa, bbbb^
ALT (U/L)	38.96 ± 3.66	135.97 ± 5.94^aaaa^	49.31 ± 8.49^a, bbbb^
AST (U/L)	36.39 ± 5.52	150.28 ± 10.24^aaaa^	49.13 ± 6.28^a, bbbb^

Statistical significance and comparisons: ^a^*p*<0.05, ^aa^*p*<0.01, ^aaa^*p*<0.001, ^aaaa^*p*<0.0001 vs. control; ^b^*p*<0.05, ^bb^*p*<0.01, ^bbb^*p*<0.001, ^bbbb^*p*<0.0001 vs. IR

*SOD *superoxide dismutase, *GSH *glutathione, *LOOH *lipid hydroperoxide, *TAS *total antioxidant status, *TOS *total oxidant status, *MDA *malondialdehyde, *MPO *myeloperoxidase, *ROS *reactive oxygen species, *ALT *alanine aminotransferase, *AST *aspartate aminotransferase

### Pro-inflammatory and anti-inflammatory cytokines and NF-κB levels

TNF-α levels (pg/100 µg protein) were 42.49 ± 1.70 in the control group, 71.65 ± 3.30 in the IR group, and 43.89 ± 1.41 in the FLX + IR group. TNF-α levels were significantly increased in the IR group compared with the control group (*p* < 0.0001). In the FLX + IR group, TNF-α levels were significantly reduced compared with the IR group (*p* < 0.0001) and did not differ significantly from those in the control group (*p* > 0.05). IL-1β levels (pg/100 µg protein) were 182.50 ± 1.61 in the control group, 127.05 ± 2.21 in the IR group, and 188.60 ± 2.71 in the FLX + IR group. IL-1β levels were significantly decreased in the IR group compared with the control group (*p* < 0.0001). In contrast, IL-1β levels were significantly increased in the FLX + IR group compared with the IR group (*p* < 0.0001) and were significantly higher than those in the control group (*p* < 0.001). IL-6 levels (pg/100 µg protein) were 103.09 ± 1.42 in the control group, 162.64 ± 3.09 in the IR group, and 116.07 ± 1.86 in the FLX + IR group. IL-6 levels were significantly elevated in the IR group compared with the control group (*p* < 0.0001). Although IL-6 levels were significantly reduced in the FLX + IR group compared with the IR group (*p* < 0.0001), they remained significantly higher than those in the control group (*p* < 0.0001). NF-κB levels (ng/100 µg protein) were 1.03 ± 0.02 in the control group, 1.63 ± 0.04 in the IR group, and 1.15 ± 0.02 in the FLX + IR group. NF-κB levels were significantly increased in the IR group compared with the control group (*p* < 0.0001). In the FLX + IR group, NF-κB levels were significantly decreased compared with the IR group (*p* < 0.0001), but remained significantly higher than those in the control group (*p* < 0.0001); (Table 2) (Fig. 4).

**Table 2:** Comparative results of pro-inflammatory and anti-inflammatory cytokines and NF-κB levels

Groups	Control group	IR group	FLX + IR group
	Pro-inflammatory and anti-inflammatory cytokines and NF-κB levels		
TNF-α (pg/100 µg protein)	42.49 ± 1.70	71.65 ± 3.30 ^aaaa^	43.89 ± 1.41 ^bbbb^
IL-1β (pg/100 µg protein)	182.50 ± 1.61	127.05 ± 2.21 ^aaaa^	188.60 ± 2.71 ^aaaa, bbbb^
IL-6 (pg/100 µg protein)	103.09 ± 1.42	162.64 ± 3.09 ^aaaa^	116.07 ± 1.86 ^aaaa, bbbb^
NF-κB (ng/100 µg protein)	1.03 ± 0.02	1.63 ± 0.04 ^aaaa^	1.15 ± 0.02 ^aaaa, bbbb^

Statistical significance and comparisons: ^a^*p*<0.05, ^aa^*p*<0.01, ^aaa^*p*<0.001, ^aaaa^*p*<0.0001 vs. control; ^b^*p*<0.05, ^bb^*p*<0.01, ^bbb^*p*<0.001, ^bbbb^*p*<0.0001 vs. IR

*TNF-α *tumor necrosis factor-alpha, *IL-1β *interleukin-1 beta,* IL-6 *interleukin-6, *NF-κB *nuclear factor kappa B

### Specific injury-related markers

NO levels (mmol/100 mg protein) were 43.61 ± 1.01 in the control group, 73.99 ± 0.71 in the IR group, and 48.68 ± 1.25 in the FLX + IR group. NO levels were significantly increased in the IR group compared with the control group (*p* < 0.0001). In the FLX + IR group, NO levels were significantly reduced compared with the IR group (*p* < 0.0001), but remained significantly higher than those in the control group (*p* < 0.0001). CASP-9 levels (ng/100 mg protein) were determined as 0.81 ± 0.04 in the control group, 1.42 ± 0.02 in the IR group, and 0.79 ± 0.02 in the FLX + IR group. CASP-9 levels were significantly elevated in the IR group compared with the control group (*p* < 0.0001). In the FLX + IR group, CASP-9 levels were significantly decreased compared with the IR group (*p* < 0.0001) and did not differ significantly from the control group (*p* > 0.05). MMP-9 levels (ng/100 mg protein) were 0.28 ± 0.03 in the control group, 0.50 ± 0.02 in the IR group, and 0.33 ± 0.01 in the FLX + IR group. MMP-9 levels were significantly higher in the IR group than in the control group (*p* < 0.0001). In the FLX + IR group, MMP-9 levels showed a modest but significant difference compared with the control group (*p* < 0.05) and were markedly reduced compared with the IR group (*p* < 0.0001). 8-OHdG levels (ng/100 mg protein) were 4.70 ± 0.13 in the control group, 8.25 ± 0.60 in the IR group, and 6.03 ± 0.18 in the FLX + IR group. A significant increase in 8-OHdG levels was observed in the IR group compared with the control group (*p* < 0.0001). In the FLX + IR group, 8-OHdG levels remained significantly higher than those in the control group (*p* < 0.0001) but were significantly lower than those in the IR group (*p* < 0.0001). HA levels (pg/100 mg protein) were 45.93 ± 1.75 in the control group, 73.68 ± 1.50 in the IR group, and 48.28 ± 1.54 in the FLX + IR group. HA levels were significantly increased in the IR group compared with the control group (*p* < 0.0001). In the FLX + IR group, HA levels were slightly but significantly higher than those in the control group (*p* < 0.05), while being markedly lower than those in the IR group (*p* < 0.0001; Table 3; Figs. 2, 3, and 5).

**Table 3:** Comparative results of specific injury-related markers

Groups	Control group	IR group	FLX + IR group
	Specific injury-related markers		
NO (mmol/100 mg protein)	43.61 ± 1.01	73.99 ± 0.71^aaaa^	48.68 ± 1.25^aaaa, bbbb^
CASPASE-9 (ng/100 mg protein)	0.81 ± 0.04	1.42 ± 0.02^aaaa^	0.79 ± 0.02^bbbb^
MMP-9 (ng/100 mg protein)	0.28 ± 0.03	0.50 ± 0.02^aaaa^	0.33 ± 0.01^a, bbbb^
8-OhdG (ng/100 mg protein)	4.70 ± 0.13	8.25 ± 0.60^aaaa^	6.03 ± 0.18^aaaa, bbbb^
HA (pg/100 mg protein)	45.93 ± 1.75	73.68 ± 1.50^aaaa^	48.28 ± 1.54^a, bbbb^

Statistical significance and comparisons: ^a^*p* < 0.05, ^aa^*p* < 0.01, ^aaa^*p* < 0.001, ^aaaa^*p* < 0.0001 vs. control; ^b^*p* < 0.05, ^bb^*p* < 0.01, ^bbb^*p* < 0.001, ^bbbb^*p* < 0.0001 vs. IR. Data are expressed as mean ± standard error of the mean (SEM)

*NO *nitric oxide, *MMP-9 *matrix metalloproteinase-9, *8-OHdG *8-hydroxy-2′-deoxyguanosine, *HA *hyaluronic acid

### Histological evaluation

**Fig. 6:**
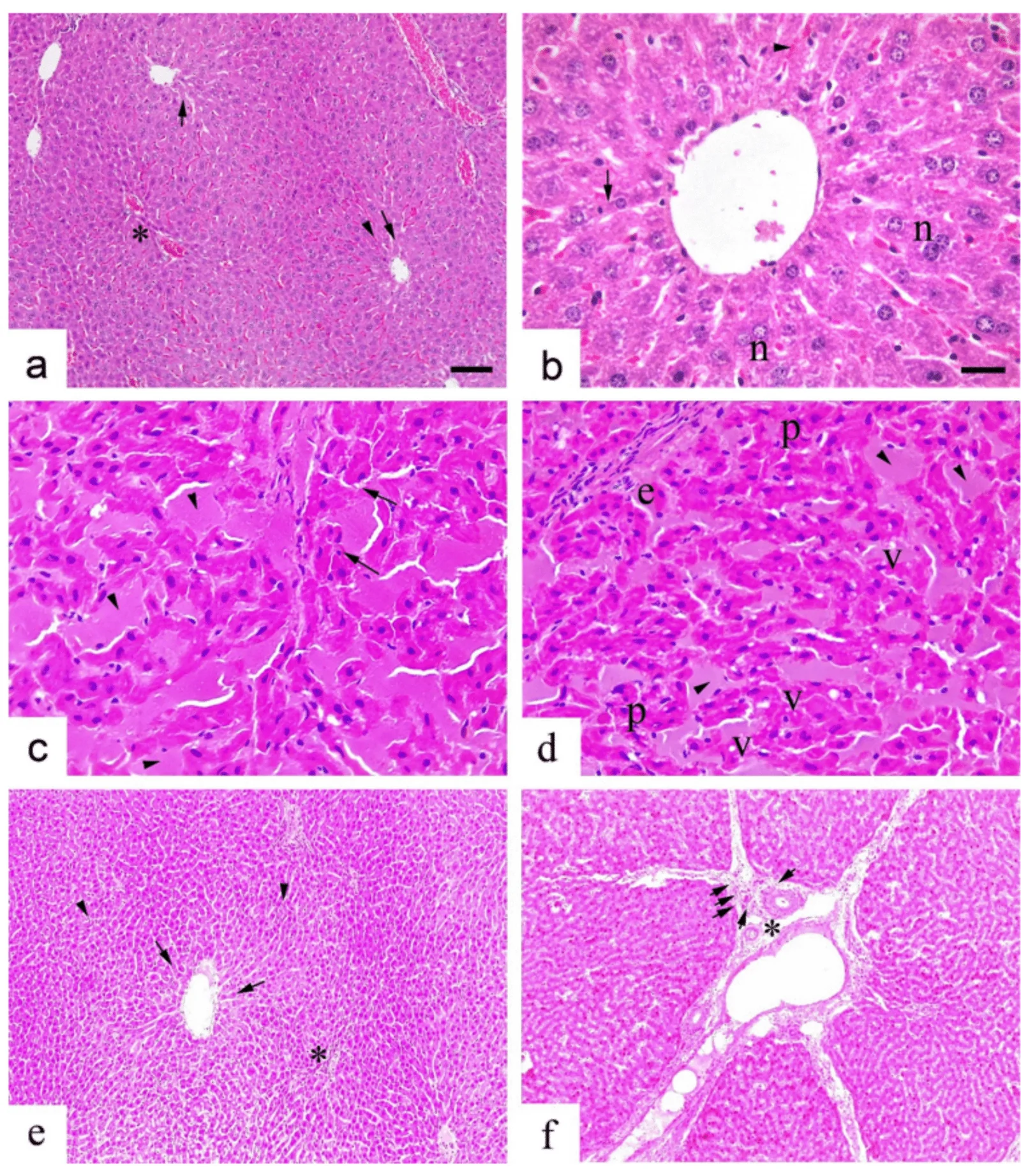
Histopathological evaluation of liver tissue in the experimental groups. C group: (**a**, left) Portal areas (asterisk), intact sinusoids (arrowhead), and well-organized hepatocyte cords extending from the central vein toward the lobular periphery (arrow); (**b**, right) normal hepatocytes with centrally located single or double nuclei (n). IR group: (**c**, left) Irregularly dilated sinusoids with fluid accumulation (arrowhead) and disorganized hepatic (Remak) cords (arrow); (**d**, right) dissociation of intercellular junctions with fluid accumulation (arrowhead), cytoplasmic hypereosinophilia and vacuolization (v) in hepatocytes, pyknotic nuclear changes (p), and neutrophil infiltration in the portal areas. FLX + IR**:**(**e**, left–f, right) Preserved hepatic cords extending from the central region toward the lobular periphery (arrow), regularly arranged sinusoids (arrowhead), and portal areas containing sparse neutrophils (asterisk). Scale bars: 25 μm (left) and 50 μm (right)

Light microscopic examination of the liver revealed that the C group exhibited a well-preserved lobular architecture, intact portal areas, and regularly arranged hepatocyte cords extending radially from the central vein toward the periphery of the lobule. The polygonal-shaped hepatocytes contained one or two centrally located nuclei. In the IR group, irregular dilatation of the sinusoids accompanied by fluid accumulation and edema was observed, along with disruption and disorganization of the hepatocyte cord continuity. This architectural distortion appeared to result, in part, from dissociation of intercellular junctions among some hepatocytes. In addition, hepatocytes demonstrated cytoplasmic hypereosinophilia and vacuolization, while nuclei exhibited dark basophilic staining and pyknotic changes. Marked neutrophil infiltration was also noted in the portal areas. In the FLX + IR group, the hepatic cord architecture was largely preserved, with regular hepatocyte alignment extending from the central vein toward the lobular periphery and maintenance of intercellular connections. In this group, the sinusoids appeared near normal, and only a limited number of neutrophils were detected within the portal areas (Fig. 6). Semi-quantitative histopathological scoring of liver injury in the experimental groups is presented in Table 4.

**Table 4:** Semi-quantitative histopathological scoring of liver injury in experimental groups

Histopathological parameters	Control group	IR group	FLX + IR group
Sinusoidal dilatation/edema	0	2	1
Disruption of hepatic cord architecture	0	2	0–1
Intercellular dissociation	0	2	0–1
Cytoplasmic hypereosinophilia/vacuolization	0	2	1
Pyknotic nuclear changes	0	1–2	0–1
Neutrophil infiltration	0	1	1
Total histopathological injury score	0	10–12	3–5

Histopathological scoring: Liver sections were semi-quantitatively evaluated for sinusoidal dilatation/edema, disruption of hepatic cord architecture, intercellular dissociation, hepatocellular degeneration, pyknotic nuclear changes, and inflammatory cell infiltration. Each parameter was graded as follows: 0 = absent/normal, 1 = mild, 2 = moderate, and 3 = severe. Total histopathological injury score was calculated as the sum of all parameter scores

## Discussion

In this study, the pathophysiological mechanisms of hepatic IR injury and the protective effects of fluoxetine were investigated. hepatic IR, commonly seen in surgery, trauma, and transplantation, involves oxidative stress, inflammation, microcirculatory dysfunction, and cell death. Ischemia deprives hepatocytes of oxygen and nutrients, while reperfusion triggers excessive reactive oxygen species (ROS) production, lipid peroxidation (LOOH, MDA), DNA damage (8-OHdG), and impaired antioxidant defenses (SOD, GSH, TAS). Oxidative stress activates NF-κB, increasing pro-inflammatory cytokines (TNF-α, IL-1β, IL-6) and neutrophil infiltration (MPO), promoting apoptosis via CASP-9 and elevating ALT and AST. Reperfusion also disrupts NO and endothelin balance, impairing microcirculation, while MMP-9 and HA reflect extracellular matrix remodeling. Fluoxetine was hypothesized to mitigate IR injury through antioxidant, anti-inflammatory, and anti-apoptotic effects, which was evaluated through biochemical and histopathological analyses to assess liver protection.

Oxidative stress, arising from an imbalance between pro-oxidant and antioxidant systems, damages lipids, proteins, and DNA, with lipid peroxidation generating MDA, which has toxic and mitogenic effects. MPO, an oxidase predominantly in polymorphonuclear leukocytes (PMNs), serves as a marker of oxidative stress and reflects PMN chemotaxis and tissue infiltration. During reperfusion, PMN infiltration amplifies ROS production, exacerbating tissue injury. 8-OHdG is a biomarker of oxidative DNA damage, indicating oxidized DNA formed under free radical influence. Excess ROS during ischemia–reperfusion triggers endothelial and leukocyte activation and cytokine release, contributing to cellular injury. Free radical generation is further enhanced via Kupffer cell and neutrophil activation, adenosine metabolism, and NO conversion to peroxynitrite. Although endogenous antioxidant systems—including SOD, catalase, glutathione peroxidase/reductase, and non-enzymatic components—attempt to limit damage, their effectiveness is diminished during reperfusion, resulting in pronounced oxidative injury (Erman et al. 2015; Yücel et al. 2019; Alak et al. 2017; Aslanoglu et al. 2024; Guner et al. 2014; Caiaffo et al. 2016; Serracino-Inglott et al. 2001; Djordjevic et al. 2011). During IR, NF-κB regulates transcription of molecules including TNF-α, ICAM-1, and inducible nitric oxide synthase (iNOS). Normally, NF-κB is inactive in the cytoplasm bound to IκB, but oxidative stress, particularly hydrogen peroxide, releases it to translocate to the nucleus and induce target genes. IR triggers two NF-κB activation phases: an early pro-inflammatory phase and a later anti-inflammatory, hepatoprotective phase, coordinating inflammation and regeneration. Hepatic injury results from microvascular obstruction and inflammation; ischemia causes energy depletion, endothelial and Kupffer cell swelling, sinusoidal narrowing, and leukocyte-platelet accumulation, producing regional hypoxia (“no-reflow”). The NF-κB and NLRP3 signaling axis has been identified as a key regulatory pathway in ischemia–reperfusion–induced inflammatory responses. NF-κB acts as an upstream transcriptional regulator controlling the expression of multiple pro-inflammatory mediators, including IL-1β, IL-6, and TNF-α. In parallel, activation of the NLRP3 inflammasome promotes caspase-1–dependent maturation and release of IL-1β and IL-18, thereby amplifying inflammatory signaling. Dysregulation of this pathway has been associated with enhanced immune cell activation and exacerbation of ischemia–reperfusion–induced tissue injury. Reperfusion increases ROS, further activating NF-κB and cytokine expression (TNF-α, IL-1, IL-6, IL-8), promoting apoptosis via caspase-3/8 and mitochondrial cytochrome c release. IL-1β amplifies TNF-α–mediated hepatocyte death and leukocyte activation. Imbalance of nitric oxide and endothelin induces vasoconstriction, worsening microcirculation and cellular oxygenation. hepatic IR injury thus arises from the interplay of oxidative stress, cytokine-mediated damage, and microvascular dysfunction (Serracino-Inglott et al. 2001; Montalvo-Jave et al. 2008; Guo et al. 2026; Konishi et al. 2017).

During hepatic IR injury, cytosolic component-containing protrusions (blebs) form on the cell membrane. These blebs may rupture and lead to the release of aminotransferases into the circulation even before overt cell death occurs. AST and ALT are among the most widely used biomarkers in both clinical diagnostics and experimental studies of liver injury, and their activities are markedly increased in acute hepatic damage (McGill et al. 2016; Panda et al. 2016). Hepatic IR injury, involving necrosis, apoptosis, and ECM alterations, is mediated by MMPs regulated by stellate, Kupffer, and inflammatory cells, leading to endothelial injury and neutrophil infiltration. MMP-9 affects ECM degradation, VEGF164 cleavage, angiogenesis, and stem cell mobilization, making MMP regulation a key therapeutic target (Wang et al. 2019; Viappiani et al. 2006; Hamada et al. 2008). Caspase-9 regulates mitochondrial stress responses and activates autophagy in hepatocytes, supporting survival and controlling inflammation. Its inhibition increases necroptosis and pro-inflammatory mediators, worsening tissue damage, suggesting caspase-9 as a potential therapeutic target in acute liver injury (Guo et al. 2016). In the liver, hyaluronic acid is mainly degraded by sinusoidal endothelial cells. Its serum levels reflect hepatocellular injury, inflammation, and stellate cell transdifferentiation, making it a valuable noninvasive biomarker for liver injury assessment (Gudowska et al. 2016).

Previous studies have demonstrated that hepatic portal occlusion results in a significant increase in MDA levels in liver tissue, accompanied by a marked decrease in SOD and GSH activities; following IR injury, ALT levels were significantly elevated in IR groups, with concomitant increases in AST levels also being observed (Deng et al. 2016). In another study, ALT, AST, and MDA levels were significantly increased, while antioxidant defenses, including SOD and GSH, were markedly reduced in the IR group (Gedik et al. 2008). In liver tissue, L-OOH levels, which serve as reliable indicators of the severity of lipid peroxidation independent of tissue oxygen tension, were reported to be increased in the IR group (Fukai et al. 2005). It has also been reported that hepatic ischemia–reperfusion injury is associated with decreased total antioxidant status (TAS) and increased TOS and MPO levels (Tokac et al. 2017; Sözen et al. 2011). In another study, MDA and TOS levels were found to be elevated in the IR group, whereas TAS and NO levels were decreased (Hekimoglu et al. 2013). Experimental hepatic ischemia–reperfusion injury has further shown that both endogenous and exogenous nitric oxide exert protective effects (Peralta et al. 2001). Serum HA levels have been shown to increase after reperfusion and to be associated with endothelial cell injury (Itasaka et al. 1995). In another study, following hepatic reperfusion, the clearance rate of serum hyaluronic acid was reduced, and serum ALT levels were higher in the IR group compared with the control group (Yoshidome et al. 2000). Increased levels of TNF-α, IL-1β, IL-6, and NF-κB have been reported in rats with hepatic ischemia–reperfusion injury (Liu et al. 2015). Similarly, in serum samples obtained from rats subjected to hepatic ischemia–reperfusion injury during liver transplantation, elevated levels of TNF-α, IL-1β, IL-6, NF-κB, ALT, and AST were observed (Liu et al. 2022). Suppression of the NF-κB signaling pathway has been shown to attenuate hepatic IR injury (Huang et al. 2019). While ischemia–reperfusion injury has been shown to increase TNF-α and caspase-9 expression in hepatocytes and vascular endothelial cells in the IR group (Durgun et al. 2022), another study also demonstrated enhanced activation of caspase-9 following IR injury (Kang et al. 2009). Increased activity of matrix metalloproteinases (MMPs) has been implicated in the pathogenesis of hepatic ischemia–reperfusion injury in ischemia–reperfusion settings. In addition, nitric oxide (NO), a key mediator of the ischemic preconditioning response, has been reported to modulate MMP activity. In particular, inducible nitric oxide synthase (iNOS)-derived NO has been shown to enhance MMP-9 expression in vascular smooth muscle cells, suggesting a functional interaction between NO signaling and MMP-mediated extracellular matrix remodeling during ischemia–reperfusion injury (Carey et al. 2004). Deficiency or specific inhibition of MMP-9 has been reported to reduce leukocyte trafficking and cytokine expression, inhibit neutrophil migration via fibronectin, and decrease MPO activation, thereby alleviating IR-induced injury (Hamada et al. 2008). Liver-specific MMP-9 inhibition preserves the VEGF–SDF-1 pathway, enhances sproc recruitment, reduces hepatic injury, and accelerates liver regeneration (Wang et al. 2019). After ischemia–reperfusion, TLR4-positive cells increase, recruiting macrophages, activating NOX2, and generating ROS, accompanied by elevated 8-OHdG, an oxidative DNA damage marker (Kimura et al. 2016). A significant correlation between the 8-OHdG index and the degree of necroinflammation has also been reported, with nuclear 8-OHdG expression observed in hepatocytes and sinusoidal cells within areas of active inflammation (Seki et al. 2002).

In this study, the IR group exhibited biochemical and functional changes indicating that hepatic IR injury arises from oxidative stress, inflammation, and cellular damage. Reduced SOD activity, decreased GSH, and diminished total antioxidant capacity suggest insufficient detoxification of reactive oxygen species during reperfusion and impaired antioxidant defenses. Elevated LOOH and MDA indicate lipid peroxidation and disrupted membrane integrity. Increased ROS and total oxidant status reflect enhanced mitochondrial dysfunction and activation of Kupffer cells and neutrophils. Elevated MPO activity further demonstrates neutrophil infiltration, amplifying tissue injury. These oxidative and inflammatory processes led to membrane blebbing and hepatocellular damage, evidenced by raised ALT and AST levels. Overall, post-reperfusion microvascular obstruction, endothelial dysfunction, and cytokine-mediated inflammation collectively contribute to hepatic parenchymal injury. Liver damage in the IR group was driven by increased ROS, weakened antioxidant defenses, lipid peroxidation, neutrophil infiltration, and consequent loss of hepatocellular integrity. The cytokine profile and NF-κB activation in the IR group indicate that hepatic ischemia–reperfusion injury is driven by the temporal and cellular dynamics of inflammation. Increased TNF-α and IL-6, together with marked NF-κB activation, suggest that ROS generated during reperfusion triggers NF-κB in endothelial cells, Kupffer cells, and infiltrating leukocytes. NF-κB nuclear translocation enhances pro-inflammatory cytokine expression, initiating acute inflammation and promoting mitochondrial dysfunction, apoptosis, and cellular injury. Elevated IL-6 supports acute-phase response and hepatic stress signaling, while increased TNF-α indicates extrinsic hepatocyte death pathways. Reduced IL-1β may reflect later-stage cytokine regulation, rapid consumption, or a preferential shift toward TNF-α and IL-6 signaling under oxidative stress. The unexpected pattern observed in IL-1β levels, characterized by a reduction in the IR group compared with controls and a subsequent increase in the FLX + IR group, may be explained by the dynamic and time-dependent nature of cytokine regulation during ischemia–reperfusion injury. IL-1β expression is known to exhibit early transient peaks followed by variable suppression depending on the severity and duration of cellular stress at the time of sampling. In the IR group, the reduction in IL-1β may reflect ischemia-induced dysfunction of cytokine-producing cells, particularly Kupffer cells, leading to impaired cytokine synthesis under severe oxidative stress conditions. In contrast, the significant increase in the FLX + IR group may be associated with fluoxetine-mediated modulation of upstream inflammatory signaling pathways, including NF-κB activation and inflammasome priming, which can enhance IL-1β production in response to ischemic injury. These findings suggest that IL-1β expression in this experimental setting is highly context-dependent and should be interpreted in conjunction with other inflammatory and oxidative stress markers rather than as a sole indicator of pro-inflammatory activity. Overall, hepatic IR injury progresses through NF-κB–mediated transcriptional reprogramming and cytokine network imbalance, establishing a self-perpetuating cycle where inflammation, cellular stress, and tissue damage reinforce each other. In the IR group, NO levels were markedly increased due to iNOS overactivation rather than physiological endothelial NO. TNF-α and IL-6 stimulate iNOS, producing excess NO that reacts with superoxide to form peroxynitrite, intensifying oxidative and nitrosative stress, mitochondrial dysfunction, and endothelial injury. Elevated caspase-9 indicates activation of intrinsic apoptosis, while increased MMP-9 reflects ECM remodeling, neutrophil infiltration, and impaired VEGF-mediated angiogenesis. Higher tissue HA levels suggest sinusoidal endothelial dysfunction and early hepatic stellate cell activation, indicating initiation of fibrotic processes. Collectively, these changes exacerbate hepatocellular damage and microvascular impairment.

Previous studies on fluoxetine’s hepatic effects focused on long-term administration, with no data on short-term effects in acute IR injury. In Danio rerio, acute fluoxetine induced liver oxidative stress, upregulated antioxidant genes (SOD, CAT, GPX), and altered ALT and ALP levels (Orozco-Hernández et al. 2023). Subchronic fluoxetine increased oxidative stress (MDA, protein carbonyl), pro-inflammatory cytokines (IL-1β, TNF-α, NF-κB), and hepatic injury markers (ALT, AST, ALP, bilirubin), while suppressing antioxidant defenses. Conversely, acute fluoxetine reduced LOOH and MDA in IR rats (Beigi et al. 2022; Guner et al. 2014). Furthermore, in ischemia–reperfusion models, short-term fluoxetine treatment has been reported to significantly attenuate the elevated levels of LOOH, MPO, TNF-α, IL-1β, and IL-6 observed in the IR group (Aksu et al. 2014). Consistent with these findings, fluoxetine treatment significantly decreased LOOH, MDA, ROS, TOS, MPO, TNF-α, IL-1β, IL-6, NF-κB, MMP-9, caspase-9, 8-OHdG, NO, and HA levels in the FLX + IR group compared with the IR group, while markedly increasing SOD, GSH, and TAS levels (Altan et al. 2023). Three weeks of fluoxetine increased serum ALT, AST, and MDA, while decreasing hepatic GSH and SOD and activating NF-κB (Zlatković et al. 2014).

In our study, in the fluoxetine-treated IR group, antioxidant defenses (SOD, GSH) were restored, while lipid peroxidation and ROS accumulation decreased. Reduced MPO activity indicates limited neutrophil infiltration, and lower ALT and AST levels reflect preserved hepatocellular integrity, demonstrating a potent, albeit partial, hepatoprotective effect in the acute phase. Fluoxetine treatment markedly reduced TNF-α and IL-6 compared with the IR group, with TNF-α approaching control levels, indicating suppression of the pro-inflammatory response. This effect is linked to inhibition of NF-κB, which was decreased but not fully normalized, allowing regenerative and cytoprotective signaling. Although NF-κB inhibition appears to play a major role in the reduction of pro-inflammatory cytokines observed in the FLX + IR group, the incomplete normalization of NF-κB levels suggests that additional upstream and parallel regulatory mechanisms may also contribute to the anti-inflammatory effects of fluoxetine. These mechanisms may include modulation of oxidative stress pathways, attenuation of ROS-mediated signaling, regulation of NLRP3 inflammasome activation, and alterations in endothelial and mitochondrial function during ischemia–reperfusion injury. IL-1β levels increased above IR and control levels, suggesting that fluoxetine modulates early cytokine release rather than fully suppressing inflammation. Controlled IL-1β signaling supports hepatic regeneration and immune reorganization. Overall, fluoxetine limits destructive inflammation while promoting a balanced cytokine profile that favors repair and adaptive responses. Fluoxetine treatment significantly reduced NO levels compared with the IR group, indicating suppression of oxidative stress–mediated iNOS activation and partial restoration of the NO–peroxynitrite balance. However, the persistence of slightly elevated NO levels suggests that physiological NO signaling involved in vasodilation and microcirculatory support is preserved. These findings are in line with previous experimental and mechanistic studies reporting that the increase in NO observed in the IR group is consistent with well-established evidence showing that hepatic I/R injury is associated with inducible nitric oxide synthase (iNOS) upregulation, leading to excessive and pathologically high NO production. This excessive NO, in the presence of elevated reactive oxygen species (ROS), contributes to the formation of peroxynitrite and subsequent oxidative and nitrosative stress, which are key mediators of cellular injury in IR models. In contrast, fluoxetine treatment significantly reduced NO levels in our study, which is most likely attributable to suppression of iNOS-derived NO production through inhibition of inflammatory signaling pathways, particularly NF-κB activation. This interpretation is supported by previous experimental studies demonstrating that fluoxetine can attenuate inflammatory responses and downregulate NF-κB-dependent mediators, including iNOS expression, thereby reducing excessive NO generation in injury models. Importantly, this reduction appears to reflect modulation of inducible NO rather than inhibition of constitutive endothelial NOS (eNOS), which is essential for maintaining microvascular perfusion and hepatic blood flow. Furthermore, serotonin signaling and SERT inhibition may indirectly influence endothelial function and NO homeostasis through platelet-derived serotonin pathways (Qin et al. 2022; Chen et al. 2013; Jedlitschky et al. 2012). Taken together, fluoxetine’s antioxidant and anti-inflammatory effects, particularly via reduction of ROS generation, are likely to play a predominant role in limiting peroxynitrite formation and restoring redox balance. While endothelial nitric oxide synthase (eNOS)-mediated physiological NO production cannot be directly assessed in the present study, the observed partial preservation of NO levels may suggest maintenance of basal nitric oxide signaling involved in microvascular homeostasis; however, this interpretation remains indirect and should be considered in the context of the measured total NO levels rather than isoform-specific activity.

Caspase-9 levels in the fluoxetine group approximated control values, indicating reduced mitochondrial stress and limitation of apoptosis, thereby supporting cellular homeostasis. Fluoxetine also significantly decreased MMP-9 levels, suggesting protection of hepatic microarchitecture by limiting inflammatory cell activation and extracellular matrix degradation, although incomplete suppression indicates ongoing repair and remodeling. Reduced 8-OHdG levels demonstrate attenuation of oxidative DNA damage, though persistent elevation compared with controls suggests that IR-induced damage is only partially reversible. Similarly, decreased HA levels indicate partial preservation of sinusoidal endothelial integrity and microcirculation, while remaining slightly above control values, reflecting continued but moderated inflammatory and fibrotic responses.

According to histopathological findings, the sham group shows orderly hepatocytes around the central vein (star) and normal sinusoidal vessels (asterisk), whereas the IR group exhibits hepatocyte degeneration, sinusoidal dilation (asterisk), vascular rupture, and marked leukocyte infiltration (Liu et al. 2015). In another study, livers from rats in the IR group demonstrated pronounced vacuolization and congestion accompanied by swollen hepatocytes (Gedik et al. 2008). After 60 min of ischemia, reperfusion caused progressive hepatic damage: pyknosis and vacuolization at 30 min, increased nuclear fragmentation and apoptotic bodies at 60 min, and widespread apoptosis at 120 min (Arab et al. 2009). It has been reported that 4 weeks of fluoxetine administration induces marked structural damage in liver tissue, including hyperemia, hemorrhage, edema, mild portal hepatitis and extensive hepatocellular necrosis. Conversely, another study demonstrated that fluoxetine largely preserved hepatic architecture (Khaksar et al. 2017; Zlatković et al. 2014).

In the present study, histological evaluation demonstrated that hepatic IR injury is characterized by oxidative stress, inflammatory cell infiltration, and microcirculatory impairment. In the IR group, irregular sinusoidal dilatation, edema, and fluid accumulation were observed, consistent with endothelial dysfunction and increased vascular permeability due to excessive ROS and NO production during reperfusion. Endothelial disruption and sinusoidal narrowing likely impaired microcirculation and promoted the “no-reflow” phenomenon, aggravating hepatocellular hypoxia and metabolic stress. Loss of hepatocyte cord continuity and disruption of intercellular junctions may result from oxidative and nitrosative lipid peroxidation, leading to membrane instability, cytoskeletal disorganization, and architectural distortion. Cytoplasmic hypereosinophilia and vacuolization indicate mitochondrial dysfunction and early necrotic changes, while pyknotic and basophilic nuclei are consistent with oxidative DNA damage and activation of mitochondrial apoptotic pathways. Prominent neutrophil infiltration within portal areas suggests NF-κB-mediated cytokine signaling and leukocyte recruitment, further amplifying tissue injury through oxidant release. In contrast, the FLX + IR group showed largely preserved hepatocyte cord architecture, suggesting that fluoxetine attenuates oxidative stress and inflammation. Reduced neutrophil infiltration and improved sinusoidal morphology indicate partial restoration of microcirculation and endothelial integrity. Overall, fluoxetine mitigated structural damage and preserved hepatic architecture, findings that strongly support the biochemical results.

**Fig. 7:**
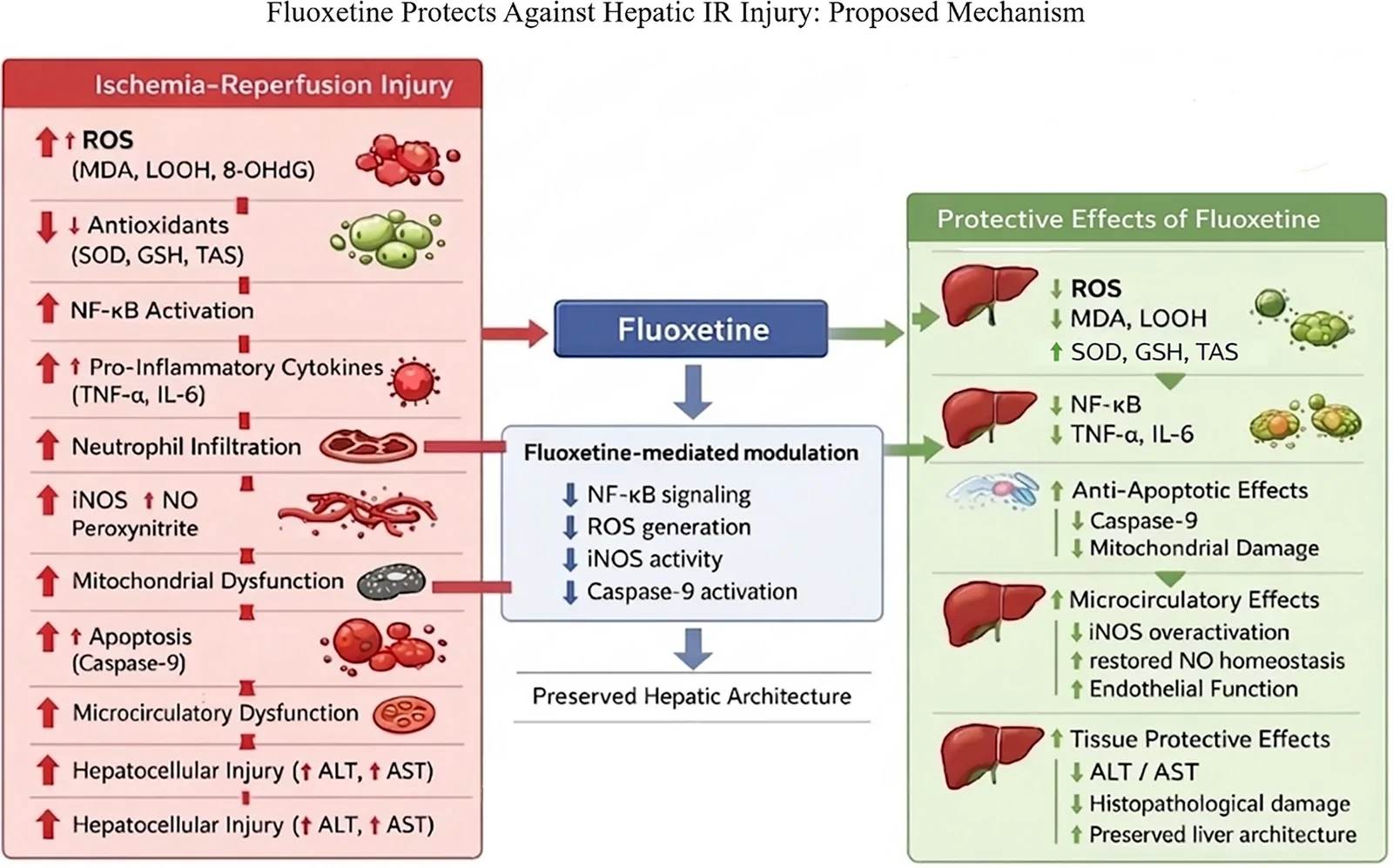
Proposed mechanism of fluoxetine-mediated protection against hepatic ischemia–reperfusion injury based on the present study findings

As illustrated in Fig. 7, hepatic IR injury is associated with excessive generation of reactive oxygen species (ROS), including increased levels of malondialdehyde (MDA), lipid hydroperoxides (LOOH), and oxidative DNA damage markers, accompanied by reduced antioxidant defenses (SOD, GSH, TAS). This imbalance leads to activation of NF-κB signaling, increased production of pro-inflammatory cytokines (TNF-α, IL-6), neutrophil infiltration, and upregulation of inducible nitric oxide synthase (iNOS), contributing to mitochondrial dysfunction, apoptosis (caspase-9 activation), and hepatocellular injury. In the present study, fluoxetine treatment significantly attenuated oxidative stress (↓MDA, ↓LOOH; ↑SOD, ↑GSH, ↑TAS), suppressed inflammatory responses (↓NF-κB, ↓TNF-α, ↓IL-6), and reduced apoptotic activity (↓caspase-9). These effects collectively contributed to improved microcirculatory function, preservation of hepatic architecture, and decreased biochemical markers of liver injury (ALT, AST). Taken together, these findings support a multi-target protective mechanism of fluoxetine involving antioxidant, anti-inflammatory, and anti-apoptotic pathways.

Although fluoxetine is primarily recognized as a selective serotonin reuptake inhibitor (SSRI), accumulating evidence suggests that its protective effects in ischemia/reperfusion injury may also involve antioxidant and anti-inflammatory mechanisms independent of classical serotonergic signaling. Previous studies have demonstrated that fluoxetine attenuates oxidative stress and suppresses NF-κB activation through regulation of the miR-450b-5p/Nrf2 axis, resulting in reduced ROS and iNOS levels together with enhanced antioxidant enzyme activity. In addition, fluoxetine has been reported to inhibit NF-κB signaling by stabilizing IκB-α, preventing IκB-α ubiquitylation, and consequently reducing the activation of NF-κB subunits p65 and p50, accompanied by decreased production of proinflammatory cytokines such as TNF-α, IL-1β, and IL-6 under ischemia/reperfusion conditions. These findings suggest that the inhibitory effect of fluoxetine on NF-κB signaling may not be solely mediated through SERT inhibition, but may also involve direct regulation of intracellular inflammatory and oxidative stress-related signaling cascades. Therefore, the protective effects observed in our hepatic I/R model may similarly be associated with both serotonergic and transporter-independent mechanisms (Qin et al. 2022; Tian et al. 2019). Nevertheless, we acknowledge that SERT expression and serotonin-related pathways were not directly evaluated in the present study.

The present study employed a preconditioning design; however, post-conditioning strategies may be more clinically relevant in settings such as liver transplantation and acute ischemic events. Future studies should therefore evaluate whether fluoxetine retains its protective efficacy when administered at the onset of reperfusion, which would further clarify its translational potential. In addition, the exclusive use of male rats may limit the generalizability of the findings, as sex-related differences can influence hepatic ischemia–reperfusion responses.

## Conclusion

Fluoxetine significantly attenuated hepatic ischemia–reperfusion injury by reducing oxidative stress, suppressing inflammatory responses, and inhibiting apoptotic pathways, thereby contributing to the preservation of hepatocellular integrity. Fluoxetine is a well-known and widely prescribed antidepressant with an established safety profile. Therefore, its potential repurposing for hepatic ischemia–reperfusion injury may have translational relevance. These findings suggest that fluoxetine may represent a promising candidate for drug repurposing in the prevention of hepatic ischemia–reperfusion injury.

## Data Availability

All data generated or analyzed during this study are available from the corresponding author upon reasonable request.
